# Editorial: Language and the digital frontier

**DOI:** 10.3389/fpsyg.2023.1305863

**Published:** 2023-11-02

**Authors:** Christopher James Hand, Sara Rodriguez-Cuadrado, Joanne Ingram

**Affiliations:** ^1^School of Education, University of Glasgow, Glasgow, United Kingdom; ^2^Department of Evolutionary and Educational Psychology, Autonomous University of Madrid, Madrid, Spain; ^3^School of Education and Social Science, University of the West of Scotland, Paisley, United Kingdom

**Keywords:** language, emoji, written language, sarcasm, emotion, natural language processing, translation

We live in an era of unprecedented global connectedness. As such, it is more crucial than ever to understand how we process language(s) and how this is shaped by—and shapes—our use of technologies.

We have collated four manuscripts from seven authors across three continents, covering diverse aspects of digital communication, including: emoji, sarcasm comprehension, emotion processing, natural language processing algorithms, and universal language.

Two papers are related to emoji. Emoji have been shown to influence both the processing of the language that they are paired with and to shape the perceptions of communicators (e.g., Hand et al., 2022). In their paper “*Sarcasm interpretation between younger and older adults*”, Cui explores a question in an emerging area, pioneered by—among others—Garcia et al. (2022). Cui's experiments investigated younger and older adults' judgments in relation to ambiguous statements accompanied by a smiling emoji. Results showed that sender age and sender–receiver relationship influenced both younger and older adults' interpretation of stimuli. For younger adults, sender age and sender–receiver relationship were significantly associated with the perceived sarcasm of emoji-based ambiguous statements. For older adults, sender age had a null effect on the interpretation of emoji-based ambiguous statements, but sender-receiver relationship impacted interpretation.

Upadhyay et al. explored how face emoji impacted the interpretation of text messages. They conducted two experiments. They found that texts paired with positive emoji were rated more positively than texts paired with negative emoji (as Boutet et al., 2021; Hand et al., 2022; Neel et al., 2023). Furthermore, texts paired with stronger-valenced emoji were rated as less neutral compared to texts paired with milder-valenced emoji. Upadhyay et al.'s 2nd experiment demonstrated that slightly positive texts paired with strong positive emoji were rated somewhat similarly to the same texts paired with mild positive emoji; however, slightly negative texts paired with strong negative emoji were rated much more negatively than the same texts paired with mild negative emoji.

Yang and Zhou investigated the acceptance of the complete English translations of *The Analects* by investigating the number of online comments, downloads, academic citations, and other factors. Based on five natural language processing (NLP) algorithms (TF-IDF, Word2Vec, GloVe, BERT, and SimHash), 15 English versions of *The Analects* were taken as samples to calculate semantic similarity. It was found that the influence of Chinese annotation on the translation semantics was great. Furthermore, different translators' identities, the translation era, the translation purpose and the translation background were not significant.

Finally, Kramer presents an article which argues for an alternative approach to written language. In their *Icono* system, “words” are represented by strings of icons. Moreover, Icono reveals sentence structure graphically before, rather than linguistically after, one begins reading. Kramer argues that using simple pictures as words would helps those with diagnoses such as dyslexia, aphasia, cerebral palsy, and autism with speech impairment.

We hope that this Research Topic stimulates debate and inspires new research.

## Author contributions

CH: Conceptualization, Project administration, Writing—original draft, Writing—review & editing. SR-C: Conceptualization, Project administration, Writing—review & editing. JI: Conceptualization, Project administration, Writing—review & editing.
